# Supplementary material for: East European sedimentary basins long heated by a fading mantle upwelling
Source: Nat Commun. 2024 May 9;15:3915. doi: 10.1038/s41467-024-48127-1 (PMC11082149; doi:10.1038/s41467-024-48127-1)
Supplement: Supplementary file 1 — Supplementary Information [file 41467_2024_48127_MOESM1_ESM.pdf]

SUPPLEMENTARY INFORMATION

East European Sedimentary Basins Long Heated by a Fading Mantle Upwelling

Alik Ismail-Zadeh[1*], Anne Davaille[2], Jean Besse[3], Yuri Volozh[4]

[1] *Karlsruhe Institute of Technology, Institute of Applied Geosciences, Karlsruhe, GERMANY*

[2] *Laboratoire FAST, CNRS and Université Paris-Saclay, Orsay, FRANCE*

[3] *Institut de Physique du Globe de Paris, Sorbonne Paris Cité, Paris, FRANCE*

[4] *MOSESTRO Exploration, Tel Aviv, ISRAEL*

* Correspondence should be sent to: alik.ismail-zadeh@kit.edu

This Supplementary Information provides an additional information on the Perm Anomaly origin (in Section SD1), magmatism within the East European platform since the Triassic times (in SD2), global plate motion models used in the study (in SD3), tectonic subsidence of sedimentary basins in the East European platform (in SD4), and the limitations, uncertainties, and sources of errors in mantle plume diffusion modeling (in SD5).

SD1. On the Perm Anomaly origin

Although our paper focuses on the mantle plume evolution associated with the Perm Anomaly (PA) over the past 200 My and it does not address the PA origin, we provide a brief overview of this topic here.

The origin of the Permo-Triassic Siberian traps is attributed to a deep mantle plume due to the source composition of the volcanic rocks, the large volume of magma erupted, and the short duration of the eruption, which was synchronous with the largest known mass-

extinction event[1-3]. Fluid dynamic experiments suggest that such a plume likely originates from a thermal boundary layer at the core-mantle boundary[4].

Reconstructing the Paleozoic mantle remains speculative due to the challenges in determining the deep mantle sources, which generate upwelling, and the paths of the upwelling within the convecting mantle. Establishing reliable Absolute Plate Motions (APMs) is further complicated by uncertainties related to tectonic deformations and continental arrangements, particularly, the complex kinematics of the North Atlantic bordering continents. Consequently, various models have been proposed including those linking the Siberian traps to the Iceland hot spot[5].

Meanwhile, the paleogeographic and palinspastic reconstruction based on paleomagnetism[6] and true polar wander-corrected paleomagnetic reconstructions[7,8] have provided evidence of a possible connection between PA and the Siberian trap province, if the source of the PA upwelling remained reasonably fixed in space. According to these reconstructions, the Siberian trap eruption site was located relatively close to PA by the end of the Permian times. Considering the mobility of the PA plume, Flament et al.[9] proposed that the PA can be linked to the Emeishan large igneous province (LIP). However, Torsvik & Domeier[10] dismissed this hypothesis citing the stability of large low-shear-velocity provinces (LLSVP), the location of the Emeishan LIP eruption site near the equator 260 My ago, and its association with the JASON LLSVP (see Fig. S1). Similarly, it is plausible to reject the notion that the PA mantle plume is linked to the Devonian Kola-Dnieper LIP (KDLIP)[11]. According to the paleogeographic and paleomagnetic reconstruction[12], the eruption sites of KDLIP were situated near the equator approximately 360 My ago and were potentially associated with the TUZO LLSVP. Therefore, KDLIP was not connected to the present PA (Fig. S1).

SD2. Magmatism of the East European platform since the Triassic times

If PA is considered to be a remnant of the old mantle plume and this plume is likely to be linked to the Siberian LIP, major magmatic events would have been associated with the Permian-Triassic boundary (the time of the Siberian traps). In the beginning of the Early Triassic, magmatism was still active in West Siberia[13]. In the Late Triassic (225-207 My) magmatism was manifested as tholeiitic and calc-alkali basalt eruptions in the Turgay basin (with the geographic centre of the basin to be around 50°N, 66°E) located to the southeast of the Urals[14,15]. However, there have been no substantial indications of volcanism recorded in

the East European (EE) lithosphere since the Early Jurassic times. A reason for that is that the EE lithosphere was thick enough to allow for magmatic eruptions. The thickness of the lithosphere exceeds 150 km in the studied area: this is visible in the S-wave velocity images of the EE lithosphere (Fig. S2) plotted using the data from Shapiro & Ritzwoller[16], and in the lithosphere-asthenosphere boundary maps based on the data from Hoggard et al.[17] (Fig. S3) and from Artemieva[18] (Fig. S4). The absence of magmatic events in the studied region of the EE platform can be explained by the thick (>150 km) lithosphere[19].

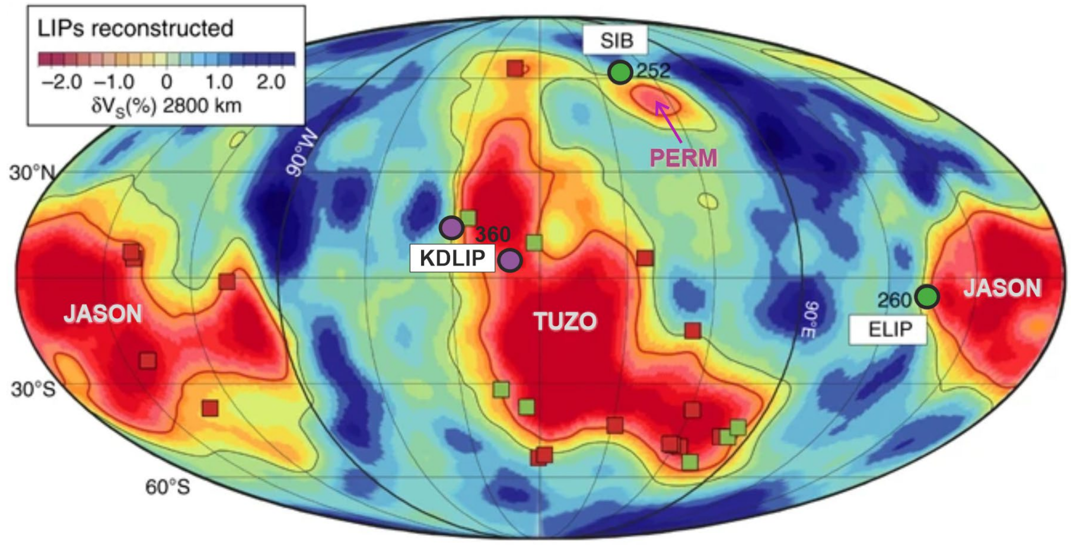

Fig. S1. Reconstruction of large igneous provinces using a hybrid reference frame[20] and draped on the tomographic model[21]. The plume generation zones marked by thick red lines in this model corresponds to the 0.9% slow S-wave seismic wave velocity contour, and the zero contours are shown as thinner black lines. LIPs with red and green symbols reconstructed with different reference frames. SIB: the 252 My old Siberian trap province; ELIP: the 260 My old Emeishan LIP; and KDLIP: the ~360 My old Kola-Dnieper LIP. KDLIP is two-component event – the Kola Alkaline Province (the left violet circle), and the Dnieper-Donets aulacogen (the right violet circle), which are located on far sides of Baltica paleocontinent[11]. The position of the KDLIP corresponds to the paleogeographic/ paleomagnetic reconstruction for 360 My (ref. 12). Modified after Torsvik & Domeier[10].

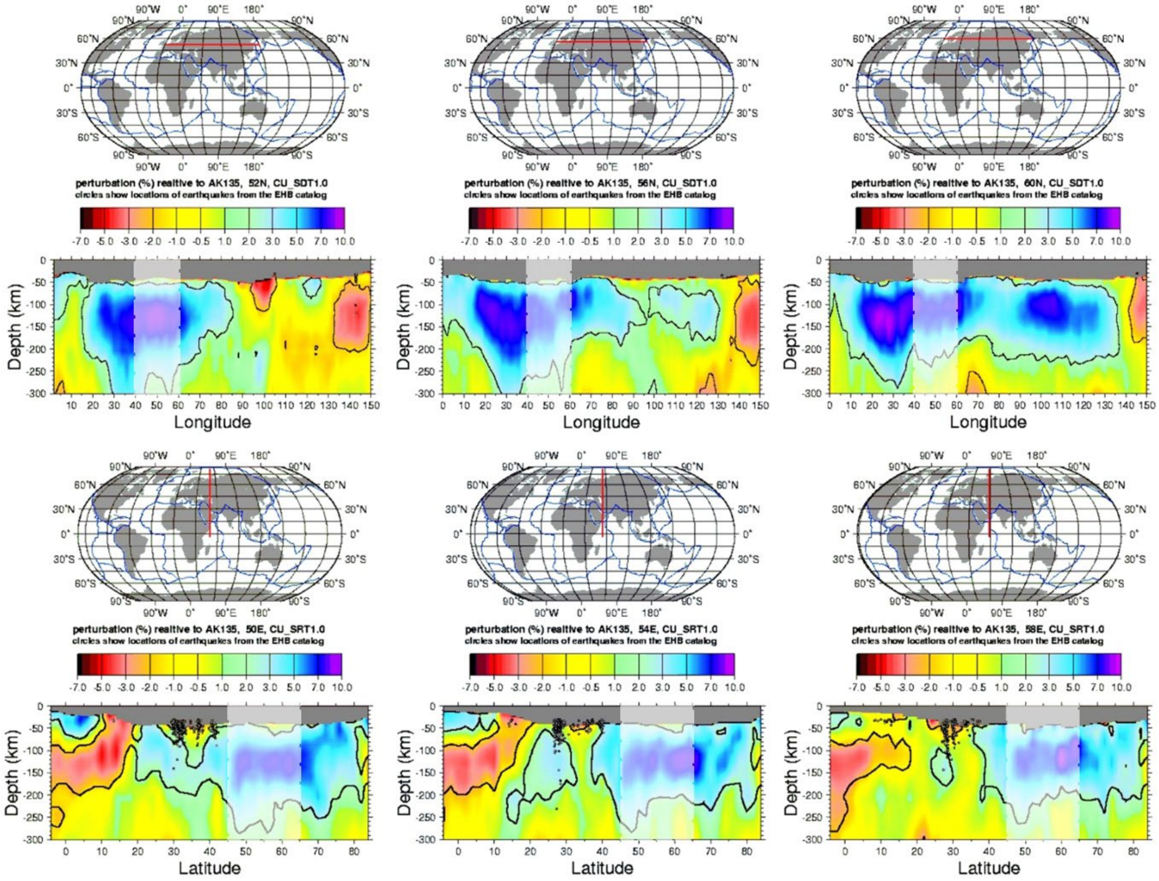

Fig. S2. Six cross-sections of S-wave velocities illustrate pronounced positive anomalies of the velocities associated with the cold and thick lithosphere of the East European platform. The cross-sections (presented as a percent deviation from the velocity in the 1-D model ak135) have been plotted using the data from the model CU_SRT1.0 (ref. 16) and the interactive plotting tool (http://ciei.colorado.edu/~nshapiro/MODEL). Black contours outline the persistent velocity anomalies, and small black dots show the earthquake locations[22]. The white shaded areas in the cross-sections correspond to the studied region. World maps above the cross sections illustrate their location (red lines).

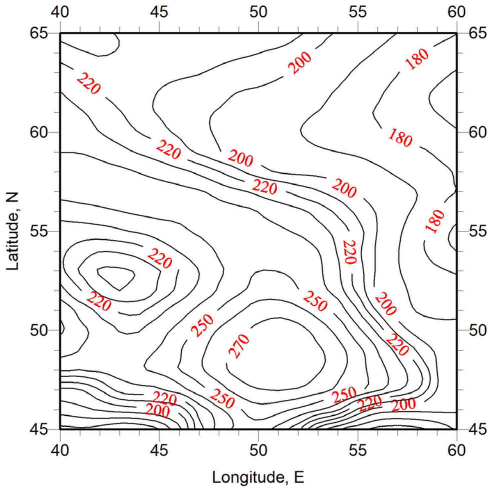

Fig. S3. The lithosphere-asthenosphere boundary (LAB) derived from the S-wave seismic velocity model[23]. Data are taken from 0.5°×0.5° grid model by Hoggard et al.[17]. Numbers on the isolines present the LAB depth here and in Fig. S4.

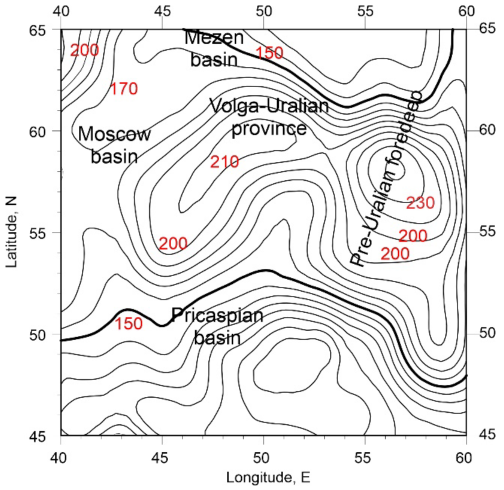

Fig. S4. The LAB defined as the intersection of the mantle geotherm with the 1300°C mantle adiabat. Data are taken from a 1°×1° grid model by Artemieva[18]. Bold lines mark the area where the LAB depth is more than 150 km.

SD3. Global plate motion models

Three models of the global lithospheric plates motion from 250 million years (My) to the present have been developed for the last decade[8,24,25]; hereinafter we refer to the models as Se2012 (ref. 24), Mü2016 (ref. 25) and Ma2016 (ref. 8). The models attempted to reconstruct the APM relative to the Earth's deep interiors and used and/or discussed the APMs from the present to 70 My – ~120 My were earlier developed[26-28]. These APMs link to the global plate circuit through Africa, as the African continent has been surrounded by mid-ocean ridges for about 170 My from the present.

The Se2012 model was based on the moving Indian–Atlantic hotspot model[27] for times earlier than 100 My, while the Ma2016 and Mü2016 models used the Torsvik et al. model[28] (reasons for that were discussed in papers[8,25]). The Torsvik et al. model[28] was preferred due to the more reasonable trench migration and net lithospheric rotation (NLR) amount implied. Indeed, their analysis suggests that APM models, in which Africa drifts NE during the 130–70 My period, are most likely in terms of trench migration rates and NLR, and also satisfies more accurately the slab remnant constraints proposed by the APM based on subductions alone[29]. A westward shift of ∼14 deg. at ∼130 My compared to the hotspot APM models falls within the range of possible fits to seismic tomography, whilst also yielding reasonable NLR and subduction zone kinematics[25].

Although reconstructions with respect to mantle are quite speculative for the Mesozoic and Palaeozoic times, three maps of global plate motion were proposed until 200 My[24], 231 My[25], and 250 My[8]. We recall that palaeomagnetic reconstructions relate the past configurations of continents to the Earth's spin axis without palaeolongitudes. Moreover, plate reconstructions with respect to mantle must include an estimate of true polar wander (TPW). Steinberger & Torsvik[30] argued that the TPW direction for the Cenozoic and Mesozoic times was largely controlled by the mass of the two antipodal large low shear-wave velocity provinces and assumed that these deep mantle bodies have been stable over a longer time scale. Hence, they proposed an estimate of TPW from 100 to 250 My.

To summarize, Seton et al.[24] used a combined reference frame: the O'Neill et al. model[27] for the last 100 My and the palaeomagnetically-derived TPW corrected reference frame[30] from 100 My to 200 My. Müller et al.[25] discussed extensively the present to 130 My APMs as mentioned above and finally used the Torsvik et al. model[28]. A close inspection of their table for the part of 130 My to 230 My is nevertheless necessary to understand what they

have done: they used a combination of a rotation due to two interpolated longitudinal shift-TPW corrections, and an additional correction using the APM rotation poles[30]. Finally, Matthews et al.[8] also used the Torsvik et al. model[28] and computed an APM between 100 My and 250 My by using the sum between three Euler poles derived from the palaeomagnetic poles[31], using TPW correction[31] and the same pole rotations as in Müller et al.[25]. These models can be inspected using the GPlates (https://www.gplates.org). Also, GPlates gives the Euler rotation parameters describing both the relative and the absolute motion of lithospheric plates with respect to what is thought to represent a mantle reference frame. We note that the plate circuit between Africa and Laurasia is identical in the two models[8,25], while it offers only minor differences with the one used by Seton et al. (ref. 24). To derive tracks of the PA upwelling related to the Se2012, Mü2016, and Ma2016 models, we have used the set of parameters presented in Table S1, where λ is the latitude, and ϕ is the longitude.

Table S1. Euler poles describing the motion of Eurasia with respect to the mantle.

	**Se2012 model**			**Mü2016 model**			**Ma2016 model**		
Age, My ago	λ, °N	ϕ, °E	angle,°	λ, °N	ϕ, °E	angle,°	λ, °N	ϕ, °E	angle,°
0	90.0	0	0	0	0	0	0	0	0
10	-38.0	39.5	1.6	-40.1	151.2	1.1	-40.1	151.2	1.1
20	-42.5	50.0	3.2	-42.5	146.7	2.3	-42.5	146.7	2.3
30	-34.0	48.0	4.2	-36.6	137.6	2.4	-36.6	137.6	2.4
40	-31.2	39.8	4.4	-27.9	115.7	1.9	-27.9	115.7	1.9
50	-13.9	47.5	6.1	-16.4	80.0	1.3	-16.4	80.0	1.3
60	17.5	68.7	5.5	-17.6	42.7	1.3	-17.6	42.7	1.3
70	32.1	65.0	6.4	3.5	78.3	2.9	3.5	78.3	2.9
80	41.7	61.7	7.8	4.7	80.1	4.9	18.6	85.8	4.6
90	50.3	48.9	11.8	12.6	75.0	8.1	29.4	81.1	8.0
100	52.4	52.1	15.5	21.7	67.2	12.0	37.3	72.5	12.7
110	51.6	68.9	22.4	31.5	64.8	15.9	52.0	65.8	16.8
120	54.2	70.0	26.5	40.7	61.6	20.9	52.2	98.4	22.8
130	50.4	70.2	30.3	38.4	62.9	24.2	54.1	70.8	25.0
140	46.3	77.0	32.3	34.1	68.0	26.6	54.6	76.2	27.7
150	45.7	69.9	34.0	33.2	60.1	28.3	54.5	71.2	29.8
160	47.4	73.6	36.8	38.2	54.2	31.2	56.2	68.8	33.5

170	50.0	73.5	39.0	46.9	55.9	31.1	55.8	72.2	36.0
180	54.6	66.7	40.1	46.0	56.1	32.5	64.1	68.6	36.3
190	51.8	49.9	44.4	42.8	44.6	36.1	66.5	64.8	37.3
200	44.4	55.2	45.0	36.7	46.9	40.3	60.5	62.3	38.9
210							52.0	57.2	40.8
220							42.2	55.6	44.0
230							36.7	55.8	46.6
240							39.8	60.2	49.2
250							43.0	64.6	51.5
260							41.6	64.4	51.1

The reconstruction of the PA's position relative to Siberia approximately 260 My ago, using the APM model[8], is depicted in Fig. S5. According to this model, the PA was situated to the south of the West Siberian Basin and is likely responsible for the Permo-Triassic volcanic activity documented in drilling operations[2]. The Late Triassic basalts in the Turgay basin could potentially mark the concluding visible volcanic activity on the surface (see also SD2), in accordance with the reconstruction presented in Fig. S5.

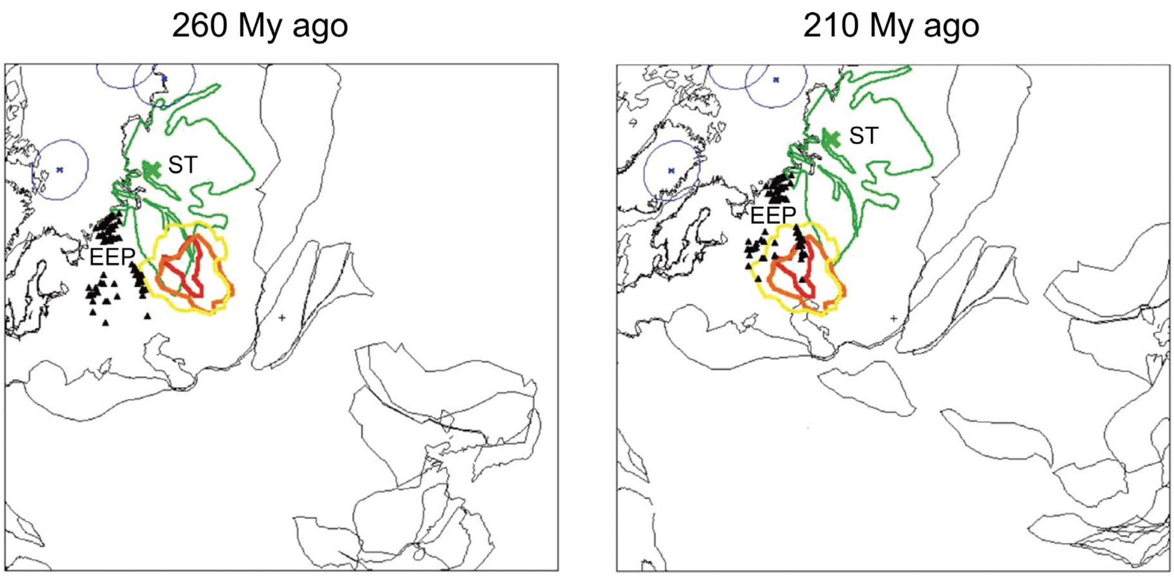

Fig. S5. Reconstruction of the PA's position relative to Siberia at the time of 260 My (Late Permian) and 210 My (Late Triassic) using the APM model[8]. Red, orange and yellow lines are the contours of the PA according to different Vs velocity anomalies. Green line marks the overall contour of the Siberian LIP. Black triangles are the oil wells in the East European platform (EEP). Blue crosses are main (present) hotspots. The green cross shows the location of Norilsk, the petrographic centre of the Siberian traps (ST); and blue crosses the present main hotspots. The absolute position of Siberia is from the APM model[8].

SD4. Tectonic subsidence of sedimentary basins in the East European platform

Sedimentary basins of the East-European (EE) platform attract considerable attention of geoscientists because of hydrocarbon deposits located there. The basins contain thick sediments ranging from about 4 km in the Moscow Basin to about 22 km in the Pricaspian basin. Many observed features in the EE basin evolution are incompatible with predictions of the stretching model as the basin subsidence often occurred in the absence of any significant stretching of the lithosphere and over time scales much longer than predicted by the model of thermal subsidence[32]. Lobkovsky et al.[33] suggested that an intraplate compression and stress-induced lithospheric deflection[34] as well as a thermal mantle upwelling followed by eclogitization-induced subsidence[35] can play an important role in the formation of EE basins.

The tectonic subsidence analysis was performed using borehole data for the Timan-Pechora basin[36], the Moscow basin[37], the Pricaspian basin[38], and the Pre-Uralian foredeep. The location of the boreholes is presented in Fig. 3 of the main text as well as in Figs. S7, S9, and S11. To determine a tectonic subsidence, the backstripping method is employed to separate isostatic effects of sediments and water loading from those of tectonic subsidence[39]. The backstripping method is based on assessments of the (Airy) isostatic response to sediment load and does not consider the flexural strength of the lithosphere, which means that any load – positive, in the case of deposition, or negative, in the case of erosion – influences the subsidence or uplift history, not just vertically below the load, but also laterally around the load. For a given sedimentary load, the magnitude of vertical deflection under the loads and the lateral wavelength of the deflection are controlled by the effective elastic thickness of the lithosphere, T_e. To evaluate the response of the elastic lithosphere to the elevation of the topography h due to sediment load, we consider the amplitude of the deflection w of the elastic lithosphere[40]:

$$w = \frac{\rho_c h}{\rho_m - \rho_c + \dfrac{E T_e^3}{12g(1-\nu^2)}\left(\dfrac{2\pi}{\lambda}\right)^4},$$

where ρ_m is the density of the mantle; ρ_c is the density of the crust; E is Young's modulus; ν is Poisson's ratio; g is the acceleration due to gravity; and λ is the topographic elevation's wavelength. The degree of the compensation $C = w / w_{\max}$ of the topographic load can be obtained as the ratio of the deflection of the elastic lithosphere to its maximum deflection

$w_{\max} = \rho_c h / (\rho_m - \rho_c)$, which reaches in the case of hydrostatic equilibrium. Considering the effective elastic thickness of the East European lithosphere to be T_e=100 km (ref. 41), E = 70 GPa, ν = 0.25, ρ_m = 3300 kg m^{-3}, ρ_c = 2800 kg m^{-3}, and g = 9.8 m s^{-2}, we can find that the topography is about 30% compensated if λ ~1000 km.

The tectonic subsidence of the EE basins since the Devonian indicates that the platform underwent two major episodes of rapid downward motion: the first episode occurred within all basins in the Late Devonian, and the second episode took place within the Timan-Pechora, Pricaspian, and Moscow basins in the Late Carboniferous/Early Permian[32]. Based on the global vertical motion of the EE platform[42], the subsidence rate of EE basins ranges from about 18 m My^{-1} in the Late Devonian (~360 My) to about 6 m My^{-1} in the Early Jurassic (~200 My). The subsidence slows down from about 4 m My^{-1} in the Middle Jurassic to 1 m My^{-1} in Pliocene (Fig. 3 in ref. 32). An increase of the subsidence rate (to about 10 m My^{-1}) can be observed in some borehole data in the Quaternary times, and the mechanism for this Quaternary subsidence acceleration in the EE basins is still poorly understood.

The **Pricaspian basin** is situated on the south-eastern portion of the East-European (EE) platform at the northern end of the Caspian Sea. The basin is approximately 600 km across from west to east; the thickness of the sedimentary cover is more than 20 km in the basin centre[43]. The basin infill is divided into three major sedimentary sequences: subsalt strata, salt, and overburden of the salt. The subsalt sequence contains Riphean through Lower Permian strata punctuated by unconformities, and it has a complex depositional history dominated by carbonate reefs and clastic fans[43]. The salt sequence consists of Kungurian (ca. 260-258 My) salt overlain by Kazanian (ca. 258-252 My) salt, which reaches a thickness of 4.5 km in the centre of the basin. The overburden of salt consists predominantly of terrigenous Upper Permian through Neogene strata[43]. The tectonic subsidence analysis[38] was performed for the central portion of the Pricaspian basin using the synthetic stratigraphic column (Fig. S6; see also Fig. 4 of the main text).

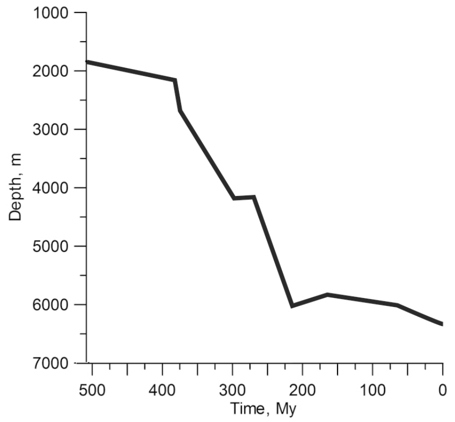

Fig. S6. Tectonic subsidence of the Pricaspian basin (data from ref. 38).

The rapid subsidence in the Pricaspian basin began in the Middle Devonian, which could be associated with the Kola-Dnieper mantle plume activity since 380 My ago[11,12]. Located to the west of the Pricaspian basin, the Dnieper-Donets aulacogen underwent stretching due to Devonian rifting[44], basaltic volcanism in the Late Devonian times[11,45], and an episode of rapid subsidence in the Late Devonian[32,46]. The Jurassic (208-170 My) uplift in the entire Pricaspian basin resulted in about 38 My hiatus.

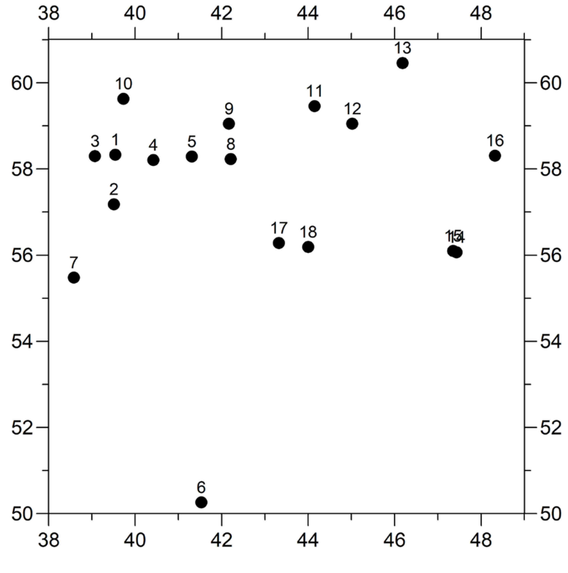

Fig. S7. Locations of the boreholes in the Moscow basin

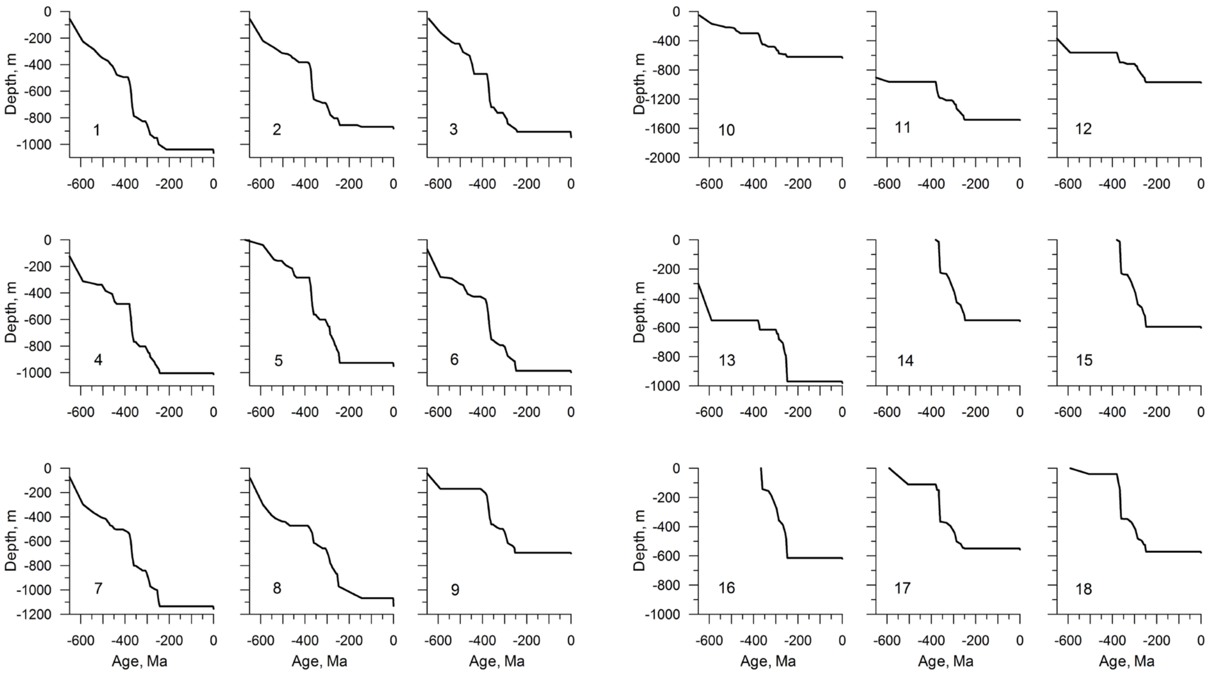

Fig. S8. Tectonic subsidence curves for the boreholes (listed in Fig. S7) drilled in the Moscow Basin (data from ref. 37)

The **Moscow Basin** is situated in the central part of the EE platform and is a typical intracratonic basin. The central part of the basin is underlain by the Riphean paleo-rift. The borders of the Moscow Basin are not clearly defined, particularly the northern boundary with the Mezen Basin. The main phase of subsidence began after the Late Vendian. In the central part of the basin the thickness of Vendian sediments exceeds 1000 m, that of Palaeozoic sediments is over 1500 m, and the thickness of Mesozoic deposits is about 500 m.

The tectonic subsidence analysis[37] was performed based on the 18 boreholes drilled in the central and southern parts of the Moscow Basin (Fig. S7; also see Fig. 3 of the main text). Subsidence in the Moscow Basin commenced in Precambrian time (Fig. S8). At least two episodes of rapid apparent subsidence since the Cambrian occurred within the Moscow Basin in the Devonian and in the Late Carboniferous/Early Permian, respectively. The Moscow Basin became an area of erosion since at least early Cretaceous.

The **Timan-Pechora Basin** is situated at the north and northeast outskirts of the EE platform and is adjacent to the Urals on the east. On the north the Timan-Pechora Basin extends beneath the Barents Sea. The main structural elements of the basin include the Timan ridge and the Pechora syneclise. The Timan ridge is a major linear basement elevation along the western edge of the basin. The Pechora syneclise is identified in the sediments and occupies most of the basin area. The maximum thickness of sediments is 7–8 km in this part

of the basin. It was formed above a zone of Palaeozoic subsidence at the eastern edge of Timan-Pechora during the orogenic phase in the Urals history.

Vertical tectonic motions of the basement of the Timan-Pechora region were studied based on an analysis of 37 exploration wells[36] (Fig. S9; also see Fig. 3 of the main text) and three lithologic-stratigraphic cross-sections along the regional seismic profiles. The tectonic subsidence curves for the wells are shown in Fig. S10. These curves display a stepwise pattern involving periods of rapid subsidence followed by phases of slower subsidence. The Timan-Pechora basin underwent tectonic uplift followed by basaltic volcanism in the Middle-Late Devonian, which could be associated with the Kola-Dnieper mantle plume. The basin experienced rapid subsidence during Late Devonian time (an episode of rifting) followed by slow subsidence of the basement (an episode of thermal relaxation). In the Early Permian the basin again experienced relatively rapid subsidence diminishing in the Middle to Late Jurassic compared to the Devonian and Permian-Triassic times. Nearly all Timan-Pechora suites became an area of erosion in the Late Cretaceous and Palaeogene.

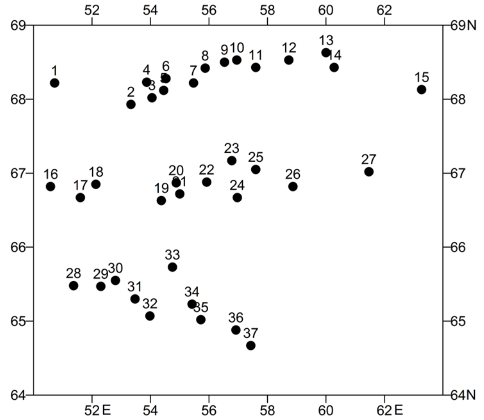

Fig. S9. The location of boreholes in the Timan-Pechora basin

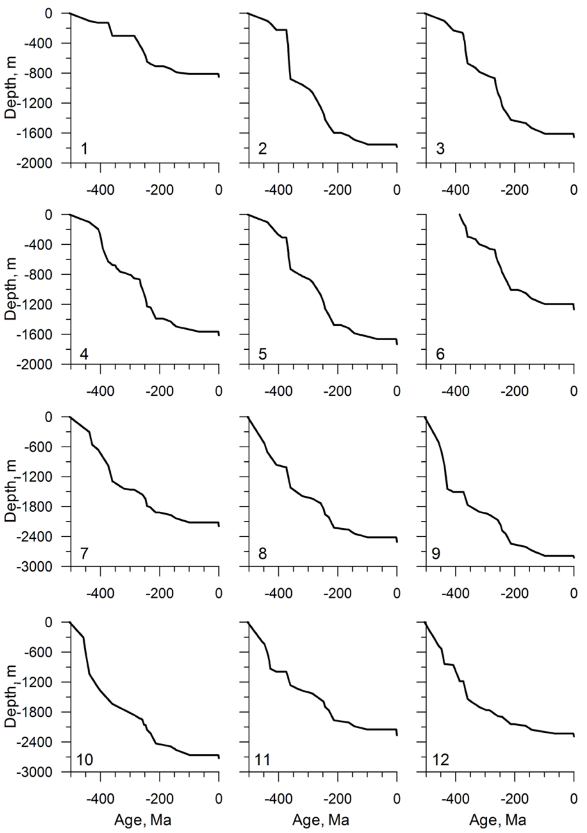

Fig. S10. Tectonic subsidence curves for the boreholes (listed in Fig. S9) drilled in the Timan-Pechora basin (data from ref. 36)

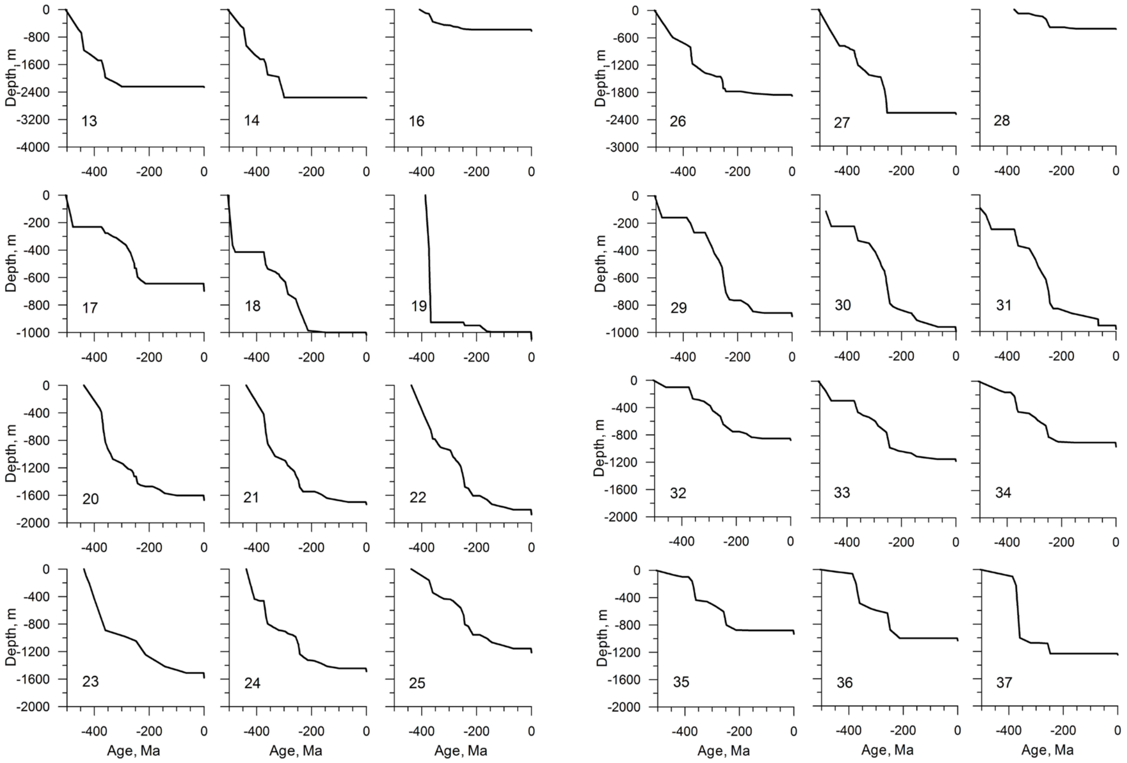

Fig. S10 (cont.)

The **Pre-Uralian depression** is expressed as a series of major depressions 10-12 km extending along the Ural-Pay Hoy folded belt. The depressions formed above a zone of Palaeozoic subsidence at the eastern edge of the Timan-Pechora plate during the orogenic phase in the Urals history. The Pre-Uralian depression extends to south towards the Pricaspian basin. 15 exploration wells (Fig. S11; see also Fig. 3 of the main text) were used to analyse the regional vertical motions. The tectonic subsidence curves (Fig. S12) show no or little subsidence for more than 100 Myr.

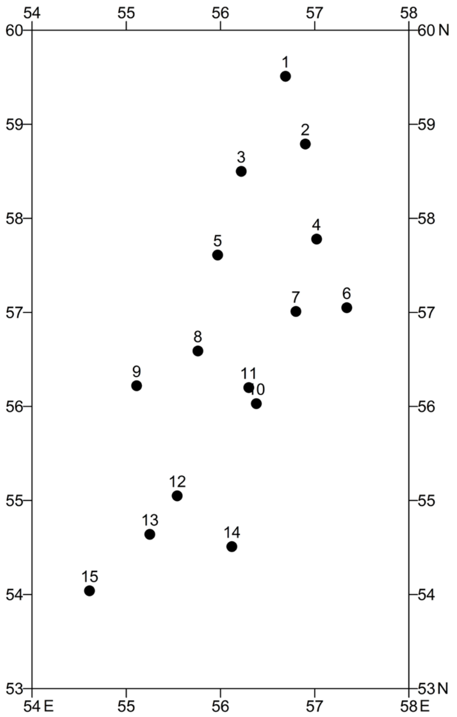

Fig. S11. The location of boreholes in the Pre-Uralian foredeep

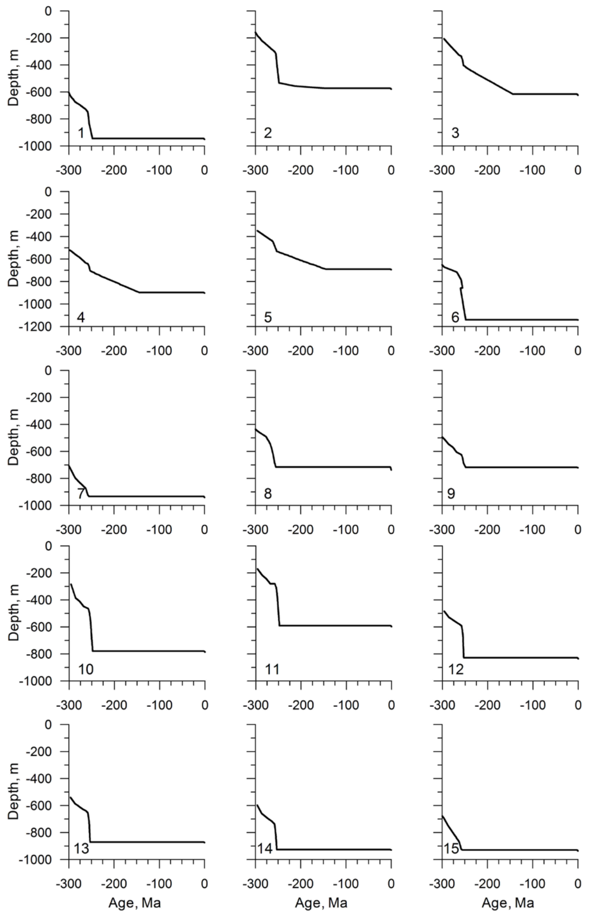

Fig. S12. Tectonic subsidence curves for the boreholes (listed in Fig. S11) in the Pre-Uralian foredeep

SD5. Mantle plume diffusion modelling: Limitations, uncertainties, and sources of errors

The mathematical model of mantle plume dynamics used in this study is simple (see *Methods* in the main text), and many complications are omitted[47]. An increase of the viscosity from the upper to the lower mantle is not considered. The adiabatic heating and cooling as well as mantle phase transformations are not included in the model, while the phase changes retard or accelerate the ascent of mantle plumes[48]. Radiative thermal conductivity in the lower mantle may lead to even faster diffusion of plume tails[49].

Numerical models of mantle plume evolution have several sources of errors and model uncertainties[47]: errors associated with the idealization of mantle plume dynamics by a set of conservation equations; errors related to the difference between the exact solution to the conservation equations and the exact solution to the discretised equations; and errors arising

from iterative solutions to the model equations. In addition, we should account for errors related to unknown mantle temperature in the geological past and the uncertainties in initial and boundary conditions of the model. Particularly, the temperature at the core-mantle boundary is unknown, it generates a source of uncertainties, and may influence model results. More detail on error sources and model uncertainties/limitations can be found in Ismail-Zadeh & Tackley[50]. While the inclusion of the model refinements and reduction of uncertainties are worthwhile, the numerical results derived from this simple model illustrate how the thermal diffusion influences the mantle plume evolution.

To assess a sensitivity of numerical results with respect to the Rayleigh number *Ra*, several numerical experiments of mantle plume evolution were performed at *Ra* ranging from about 10^4 to 10^6 (ref. 47). The experiments showed a similar pattern of the plume's decay, although the morphology of the plumes and the time of the decay were different. To reduce uncertainties in numerical models of mantle plume evolution, which are related to the initial conditions in the geological past, the present data can be assimilated to reconstruct the evolution of a present diffused mantle plume to its prominent state in the past[47,51]. With the development of modern data assimilation techniques[52] and high-resolution geophysical data acquisition, the detailed dynamic reconstruction of a prominent mantle plume from its present diffused state should become possible in the near feature.

A source of errors can arise from the geometry of a model domain, for example, a three-dimensional Cartesian model versus a spherical shell model. A detailed comparison of both models of the thermal convection showed that the mean temperature in each model attains almost the same value at the dimensionless domain size of 3×3×1 and at Rayleigh numbers greater than 10^5 (ref. 53). The dimensionless size of the model domain used in this study is 3×3×1, and $Ra = 9.5 \times 10^5$.

Also, in this paper, we do not analyse a detailed interaction of the continental lithosphere with the underlying mantle. It has been shown that a continental rheological structure and intra-plate stresses may influence the topography of the lithosphere above the mantle plume[54] and hence the thermal evolution of the lithosphere.

References

1 Campbell, I. A., Czamanske, G. K., Fedorenko, V. A., Hill, R. I. & Stepanov, V. Synchronism of the Siberian traps and the Permian–Triassic boundary. *Science* **258**, 1760–1763 (1992).

2 Saunders, A. D., England, R.W., Reichow, M.K. & White, R.V. A mantle plume origin for the Siberian traps: uplift and extension in the West Siberian Basin, Russia. *Lithos* **79**, 407–424 (2005).

3 Sobolev, S. V., Sobolev, A. V., Kuzmin, D. V., Krivolutskaya, N. A., Petrunin, A. G., Arndt, N. T., Radko, V. A. & Vasiliev, Y. R. Linking mantle plumes, large igneous provinces and environmental catastrophes. *Nature* **477**, 312–316 (2011).

4 Davaille, A. Simultaneous generation of hotspots and superswells by convection in a heterogeneous planetary mantle. *Nature* **402**, 756–760 (1999).

5 Lawver, L.A., Grantz, A. & Gahagan, L.M. Plate kinematic evolution of the present Arctic region since the Ordovician. In *Tectonic Evolution of the Bering Shelf - Chukchi Sea - Arctic Margin and Adjacent Landmasses,* Miller, E.L. Grantz, A. & Klemperer S.L., eds., pp. 333-358 (Geological Society of America Special Paper, Boulder, 2002).

6 Kazmin, V.G. & Natapov, L.M. The Paleogeographic Atlas of Northern Eurasia. 26 maps: Devonian to Neogene (380.8–6.7 Ma). TOO Institute of Tectonics of Lithospheric Plates, Moscow (1998).

7 Torsvik, T.H., Smethurst, M.A., Burke, K. & Steinberger, B. Large igneous provinces generated from the margins of the large low-velocity provinces in the deep mantle. *Geophys. J. Intern* **167**, 1447–1460 (2006).

8 Matthews, K. J., Maloney, K. T., Zahirovic, S., Williams, S. E., Seton, M. & Müller, R. D. Global plate boundary evolution and kinematics since the late Paleozoic. *Global Planet. Change* **146**, 226–250 (2016).

9 Flament, N., Williams, S., Muller, R.D., Gurnis, M. & Bower, D.J. Origin and evolution of the deep thermochemical structure beneath Eurasia. *Nat. Commun.* **8**, 14164, https://doi.org/10.1038/ncomms14164 (2017).

10 Torsvik, T.H. & Domeier, M. Correspondence: Numerical modelling of the PERM anomaly and the Emeishan large igneous province. *Nat. Commun.* **8**, 821, https://doi.org/10.1038/s41467-017-00125-2 (2017).

11 Ernst, R.E. *Large Igneous Provinces* (Cambridge University Press, Cambridge, 2014).

12 Kuzmin, M.I., Yarmolyuk, V.V., and Kravchinsky, V.A. Phanerozoic hot spot traces and paleogeographic reconstructions of the Siberian continent based on interaction with the African large low shear velocity province. *Earth Sci. Rev.* **102**, 29–59 (2010).

13 Nikishin, A.M., Ziegler, P.A., Abbott, D., Brunet, M.-F. & Cloetingh, S. Permo-Triassic intraplate magmatism and rifting in Eurasia: implications for mantle plumes and mantle dynamics. *Tectonophysics* **351**, 3 –39 (2002).

14 Bochkarev, V. S. Triassic volcanogenic rocks of the western Siberia (in Russian). In: *Triassic West Siberia*, Novosibirsk, SNIIGGMS, pp. 70-79 (2001).

15 Bochkarev, V. S., Brekhuntsov, A. M. & Deschenya, N. P. Fundamental problems of stratigraphy of the Mesozoic oil-and-gas-bearing deposits (in Russian). *Geology of Oil and Gas* **1**, 2-13 (2000).

16 Shapiro, N.M. & Ritzwoller, M.H. Monte-Carlo inversion for a global shear velocity model of the crust and upper mantle, *Geophys. J. Int.* **151**, 88–105 (2002).

17 Hoggard, M.J., Czarnota, K., Richards, F.D. et al. Global distribution of sediment-hosted metals controlled by craton edge stability. *Nat. Geosci*. **13**, 504–510 (2020).

18 Artemieva, I.M. Global 1°×1° thermal model TC1 for the continental lithosphere: Implications for lithosphere secular evolution. *Tectonophysics* **416**, 245–277 (2006).

19 Davies, D., Rawlinson, N., Iaffaldano, G. et al. Lithospheric controls on magma composition along Earth's longest continental hotspot track. *Nature* **525**, 511–514 (2015).

20 Torsvik, T.H., Steinberger, B., Ashwal, L.D., Doubrovine, P.V. & Trønnes, R. C. Earth evolution and dynamics–a tribute to Kevin Burke. *Can. J. Earth Sci.* **53**, 1073–1087 (2016).

21 Doubrovine, P. V., Steinberger, B. & Torsvik, T. H. A failure to reject: testing the correlation between large igneous provinces and deep mantle structures with EDF statistics. *Geochem. Geophys. Geosyst.* **17**, 1130–1163 (2016).

22 Engdahl, E.R., van der Hilst, R. & Buland, R. Global teleseismic earthquake relocation with improved traveltime and procedures for depth determination. *Bull. Seism. Soc. Am.* **88**, 722–743 (1998).

23 Schaeffer, A. J. & Lebedev, S. Global shear speed structure of the upper mantle and transition zone. *Geophys. J. Int*. **194**, 417–449 (2013).

24 Seton, M. R.D. Müller, R.D., Zahirovic, S., Gaina, C., Torsvik, T., Shephard, G., Talsma, A., Gurnis, M., Turner, M., Maus, S. & Chan, M. Global continental and ocean basin reconstructions since 200 Ma. *Earth Sci. Rev.* **113**, 212–270 (2012).

25 Müller, R. D., Seton, M., Zahirovic, S., Williams, S.E., Matthews, K.J., Wright, N.M., Shephard, G.E., Maloney, K.T., Barnett-Moore, N., Hosseinpour, M., Bower, D.J. & Cannon, J. Ocean basin evolution and global-scale plate reorganization events since Pangea breakup. *Ann. Rev. Earth Planet. Sci.* **44**, 107–138, doi: 10.1146/annurev-earth-060115-012211 (2016).

26 Müller, R. D., Royer, J. Y. & Lawver, L. A. Revised plate motions relative to the hotspots from combined Atlantic and Indian Ocean hotspot tracks. *Geology* **21**, 275–278 (1993).

27 O'Neill, C., Müller, D. & Steinberger, B. On the uncertainties in hot spot reconstructions and the significance of moving hot spot reference frames. *Geochem. Geophys. Geosyst.* **6**(4), Q04003, doi:10.1029/2004GC000784 (2005).

28 Torsvik, T.H., Steinberger, B., Cock, L.R.M., Burke, K. Longitude: Linking Earth's ancient surface to its deep interior. *Earth Planet. Sci. Lett.* **276**, 273–282 (2008).

29 van der Meer, D. G., Spakman, W., van Hinsbergen, D. J. J., Amaru, M. L. & Torsvik, T. H. Towards absolute plate motions constrained by lower-mantle slab remnants. *Nat. Geosci.* **3**(1), 36–40 (2010).

30 Steinberger, B. & Torsvik, T. H. Absolute plate motions and true polar wander in the absence of hotspot tracks. *Nature* **452**, 620–623 (2008).

31 Torsvik, T.H., Van der Voo, R., Preeden, U., Mac Niocaill, C., Steinberger, B., Doubrovine, P.V. et al. Phanerozoic polar wander, paleogeography and dynamics. *Earth Sci. Rev.* **114** (3-4), 325–368 (2012).

32 Ismail-Zadeh, A.T. The Devonian to Permian subsidence mechanisms in basins of the East-European platform. *J. Geodyn.* **26**, 69–83 (1998).

33 Lobkovsky, L.I., Cloetingh, S., Nikishin A.M., Volozh, Yu.A., Lankreijer, A.C., Belyakov S.L. et al. Extensional basins of the former Soviet Union – structure, basin formation mechanisms and subsidence history. *Tectonophysics* **266**, 251–286 (1996).

34 Cloetingh, S. Intraplate stresses: A new tectonic element in basin analysis. In *New Perspectives in Basin Analysis*, Kleinspehn, K.L. & Paola, C., eds., pp. 205–230. (Springer-Verlag, New-York, 1988).

35 Ismail-Zadeh, A.T., Naimark B. M. and Lobkovsky L. I. Hydrodynamic model of sedimentary basin formation based on development and subsequent phase transformation of a magmatic lens in the upper mantle. *Comput. Seism. Geodyn.* **3**, 42–53 (1996).

36 Ismail-Zadeh, A. T., Kostyuchenko, S. L. & Naimark, B. M. The Timan-Pechora Basin (northeastern European Russia): tectonic subsidence analysis and a model of formation mechanism. *Tectonophysics* **283**, 205–218 (1997).

37 Kostyuchenko, S. L. & Ismail-Zadeh, A. T. The origin of rapid phases of subsidence and sedimentation in the Moscow syneclise on the basis of deep seismic studies and quantitative analysis (in Russian). *Exploration and Preservation of Resources (Razvedka i Okhrana Nedr)* **2**, 37–40 (1998).

38 Brunet, M.-F., Volozh, Y. A., Antipov, M. P. & Lobkovsky, L. I. The geodynamic evolution of the Precaspian Basin (Kazakhstan) along a north-south section. *Tectonophysics* **313**, 85–106 (1999).

39 Steckler, M.S. & Watts, A.B. Subsidence of the Atlantic type continental margin off New York. *Earth Planet. Sci. Lett.* **42**, 1–13 (1978).

40 Turcotte, D.L. & Schubert, G. *Geodynamics* 2nd ed. (Cambridge University Press, Cambridge, 2002).

41 Kaban, M. K., Chen, B., Tesauro, M., Petrunin, A. G., El Khrepy, S. & Al-Arifi, N. Reconsidering effective elastic thickness estimates by incorporating the effect of sediments: A case study for Europe. *Geophys. Res. Lett.* **45**(18), 9523–9532 (2018).

42 Nalivkin, V.D. & Yakobson, K.E., eds. *Geological Structure of the USSR and Pattern of Mineral Resources. Vol. 1. Russian Platform* (Nedra, Leningrad, 1985).

43 Volozh, Y. A., Talbot, C. J. & Ismail-Zadeh, A. T. Salt structures and hydrocarbons in the Pricaspian basin. *Amer. Assoc. Petrol. Geol. Bull.* **87**, 313–334 (2003).

44 Stovba, S., Stephenson, R.A. & Kivshik, M. Structural features and evolution of the Dnieper-Donets Basin, Ukraine, from regional seismic reflection profiles. *Tectonophysics* **268**, 127–148 (1996).

45 Wilson, M. & Lyashkevich, Z.M. Magmatism and the geodynamics of rifting of the Pripyat-Dnieper-Donets rift, East European Platform. *Tectonophysics* **268**(1-4), 65–81 (1996).

46 Lobkovsky, L.I., Ismail-Zadeh, A.T., Krasovsky, S.S., Kuprienko, E.Ya. & Cloetingh, S. Gravity anomalies and possible formation mechanism of the Dnieper-Donets Basin. *Tectonophysics* **268**, 281–292 (1996).

47 Ismail-Zadeh, A., Schubert, G., Tsepelev, I. & Korotkii, A. Three-dimensional forward and backward numerical modeling of mantle plume evolution: Effects of thermal diffusion. *J. Geophys. Res.* **111**, B06401, doi:10.1029/2005JB003782 (2006).

48 Honda, S., Yuen, D.A., Balachandar, S. & Reuteler, D. Three dimensional instabilities of mantle convection with multiple phase transitions. *Science* **259**, 1308–1311 (1993).

49 Badro, J., Rueff, J.-P., Vanko, G., Monaco, G., Figuet, G. & Guyot, F. Electronic transitions in perovskite: Possible nonconvecting layers in the lower mantle. *Science* **305**, 383–386 (2004).

50 Ismail-Zadeh, A. & Tackley, P.J. *Computational Methods for Geodynamics* (Cambridge University Press, Cambridge, 2010).

51 Ismail-Zadeh, A., Schubert, G., Tsepelev, I. & Korotkii, A. Inverse problem of thermal convection: Numerical approach and application to mantle plume restoration *Phys. Earth Planet. Inter.* **145**, 99-114 (2004).

52 Ismail-Zadeh, A., Castelli, F., Jones, D. & Sanchez, S., eds. *Applications of Data Assimilation and Inverse Problems in the Earth Sciences* (Cambridge University Press, Cambridge, 2023).

53 O'Farrell, K.A., Lowman, J.P. & Bunge, H.-P. Comparison of spherical-shell and plane-layer mantle convection thermal structure in viscously stratified models with mixed-mode heating: implications for the incorporation of temperature-dependent parameters. *Geophys. J. Inter.* **192**(2), 456–472 (2013).

54 Burov, E. & Gerya, T. Asymmetric three-dimensional topography over mantle plumes. *Nature* **513**, 85–89 (2014).
